# SENP1 Is a Crucial Regulator for Cell Senescence through DeSUMOylation of Bmi1

**DOI:** 10.1038/srep34099

**Published:** 2016-09-23

**Authors:** Nansong Xia, Juan Cai, Feifei Wang, Baijun Dong, Song Liu, Fengling Chen, Jinke Cheng, Yong Zuo

**Affiliations:** 1 Department of Biochemistry and Molecular Cell Biology, Shanghai Key Laboratory for Tumor Microenvironment and Inflammation, Shanghai Jiao Tong University School of Medicine, Shanghai, China; 2 Department of Urology, Renji Hospital, Shanghai Jiao Tong University School of Medicine, Shanghai, China; 3 Department of Respiratory Medicine, Xinhua Hospital, Shanghai Jiao Tong University School of Medicine, Shanghai, China; 4 Shanghai Third People’s Hospital, Shanghai Jiao Tong University School of Medicine, Shanghai, China

## Abstract

Cell senescence can limit proliferative potential and prevent tumorigenesis. Bmi1 is a key regulator in cell senescence by suppressing the Ink4a/Arf locus. However, how to regulate Bmi1 activity in cell senescence is largely unknown. Here, we show that SENP1 plays an important role in cell senescence by regulating Bmi1 SUMOylation. *Senp1*^−/−^ primary MEF cells show resistance to cell senescence induced by passaging or other senescence inducing signals. SENP1 deficiency also reduces oncogene H-Ras^V12^-induced senescence, and enhances H-Ras^V12^-induced cell transformation. We further show that in *Senp1*^−/−^ MEFs the expression of p19^Arf^, an important regulator in p53/p21-mediated cell senescence, is markedly reduced. Meanwhile, we demonstrate that SENP1 can specifically de-SUMOylate Bmi1 and thereby decreases the occupancy of Bmi1 on p19^Arf^ promoter leading to decrease of H2AK119 mono-ubiquitination and up-expression of p19^Arf^. These data reveal a crucial role of SENP1 in regulation of cell senescence as well as cell transformation.

Cell senescence is an irreversible growth-arrested status that is characterized by flat cell morphology, senescence-associated-β-galactosidase (SA-β-gal) activity, and up-regulation of cell cycle inhibitors^1^. A variety of stresses, including telomere uncapping, DNA damage, oxidative stress, and oncogene can trigger cell senescence^2^. Senescence triggered by cell-intrinsic mechanisms, such as DNA damage and oxidative stress, are referred to as replicative senescence, whereas senescence induced by ectopic expression of activated onco-proteins, such as Ras and Raf, as oncogene-induced senescence (OIS)^3^,^4^,^5^. Cell senescence plays an important role in tumor suppression and organ aging^2^,^6^,^7^,^8^. Different stress signals induce cell senescence through activation of p16^Ink4a^/Rb or p19^Arf^/p53 pathway^2^,^9^,^10^,^11^.

PcG complexes are a family of transcriptional repressors and suppress the expression of lots of genes including p16^Ink4a^ or p19^Arf^ via modulation of histone modification. PcG has two multi-protein complexes, polycomb repressive complex 1 (PRC1) and 2 (PRC2). PRC1 includes Bmi1, CBXs, PHC1-3, RNF1-2, and SCML1-2 proteins^12^. PRC1 mono-ubiquitinates histone H2A at lysine 119 (H2AK119ub)^13^,^14^,^15^, which is associated with gene silencing^16^,^17^,^18^. PRC2 contains EZH2, EED, and SUZ12, which trimethylates histone H3 on Lys 27 (H3K27me3)^19^,^20^. It has been reported that PRC1 components Bmi1, CBX7, and CBX8 can delay the onset of senescence in mouse and human embryonic fibroblasts by repressing the expression of p16^Ink4a^ or p19^Arf^ 21,22,23.

SUMOylation has emerged as an important protein modification in regulation of many cellular processes^24^. SUMOylation is a dynamic process, which is sequentially catalyzed by SUMO-specific E1, E2 and E3 ligase and can be reversed by SUMO-specific proteases (SENPs)^25^,^26^. Recently, it was reported that DNA damage induces SUMOylation of Bmi1^27^. SUMOylation can regulate Bmi1 to be recruited to DNA breaks. However, whether SUMOylation modulates Bmi1 activity in cell senescence is largely unknown. In this study, we show that *Senp1*^−/−^ primary MEF cells are resistant to cell senescence triggered by passaging or other senescence inducing signals. Moreover, *Senp1*^−/−^ MEF cells are also resistant to oncogene H-Ras^V12^-induced senescence and show enhanced H-Ras^V12^-induced cell transformation. Mechanistically, SENP1 de-SUMOylates Bmi1, and thus decreases the occupancy of Bmi1 on p19^Arf^ promoter locus leading to the expression of p19^Arf^/p53, and subsequently promotes cell senescence.

## Results

### SENP1 deficiency reduces the senescence of MEF cells

**Figure 1:**
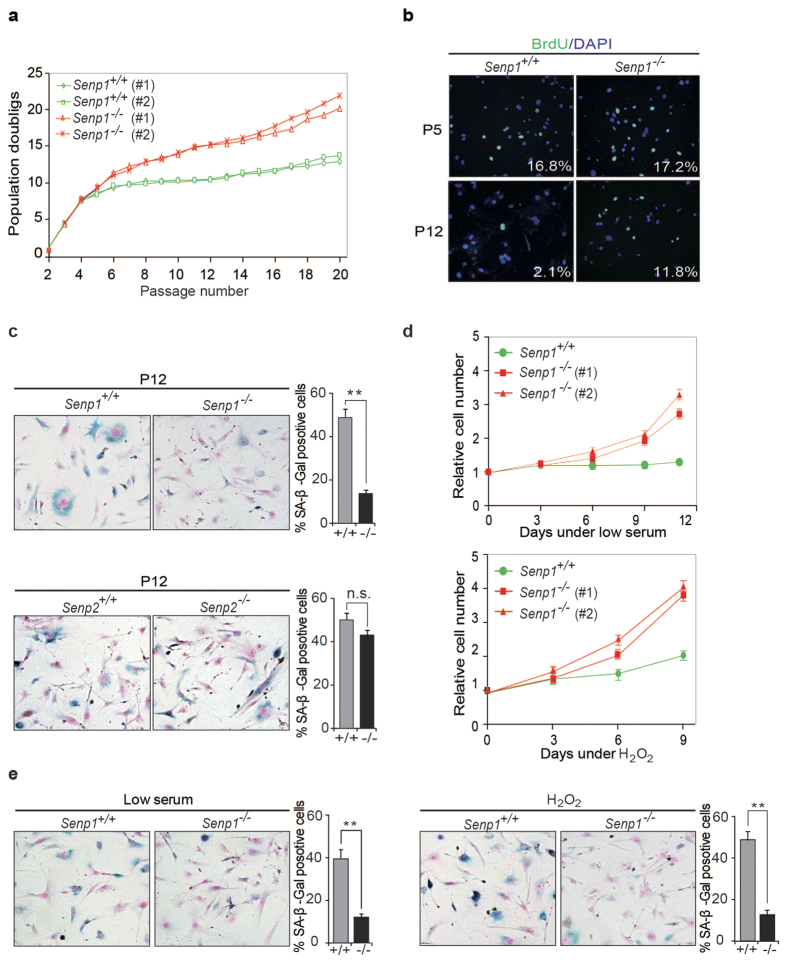
*Senp1*^−/−^ MEF cells are resistant to cellular senescence. (**a**) Population doublings (PD) over serial passaging according to a 3T3 protocol were measured in *Senp1*^+/+^ and *Senp1*^−/−^ cell lines. (**b**) Proliferation rate of primary MEF cells at passage 5 (P5) and passage 12 (P12) were measured by using BrdU labelling assay. (**c**) *Senp1*^+/+^*, Senp1*^−/−^*, Senp2*^+/+^ *and Senp2*^−/−^ MEF cells at passage 12 (P12) were stained for SA-β-Gal activity. Representative images are shown in left panel. SA-β-Gal positive cells were counted at least in five fields. Data represent the mean ± S.E.M. of three independent experiments (right panel) (**P < 0.01, Student’s t-test). (**d**) Cell proliferation rates of *Senp1*^+/+^ and *Senp1*^−/−^ MEF cells were measured under the conditions of low serum (3%) (upper) or sub-lethal concentration of H_2_O_2_ (50 μM) (lower). (**e**) *Senp1*^+/+^ and *Senp1*^−/−^ MEF cells in low serum or 50 μM H_2_O_2_ were stained for SA-β-Gal activity. Data represent the mean ± S.E.M. of three independent experiments (**P < 0.01, Student’s t-test).

Our previous study has demonstrated that *Senp1*^−/−^ MEF cells were easily immortalized by using a 3T3 protocol compared to wild-type (WT) cells, indicating that SENP1 deficiency might keep MEFs away from cell senescence. To test it, we freshly isolated MEF cells from *Senp1*^+/+^ and *Senp1*^−/−^ embryos at E12.5 and cultured them by using a 3T3 protocol. As shown in Fig. 1a, *Senp1*^+/+^ MEF cells entered growth plateau phase at 6 to 8 passages, whereas *Senp1*^−/−^ MEF cells proliferated steadily up to 20 passages. Consistent with this observation, *Senp1*^−/−^ MEF cells exhibited a higher BrdU incorporation rate than *Senp1*^+/+^ MEF cells did (Fig. 1b), indicating higher proliferation rate in *Senp1*^−/−^ MEF cells. We further stained SA-β-gal and found that SA-β-gal positive cells were markedly less in *Senp1*^−/−^ but not in *Senp2*^−/−^ MEF cells when compared with control WT MEF cells (Fig. 1c). Furthermore, we determined whether SENP1 deficiency could affect the senescence induced by serum deprivation or oxidative stress. *Senp1*^+/+^ and *Senp1*^−/−^ MEF cells at passage 5 were cultured in medium containing 50 μM H_2_O_2_ or 3% serum. Both conditions reduced the proliferation of *Senp1*^+/+^ MEFs but not *Senp1*^−/−^ cells (Fig. 1d). We also confirmed that SENP1 deficiency could decrease the senescence in both conditions (Fig. 1e).

### SENP1 deficiency decreases H-Ras^V12^-induced senescence

**Figure 2:**
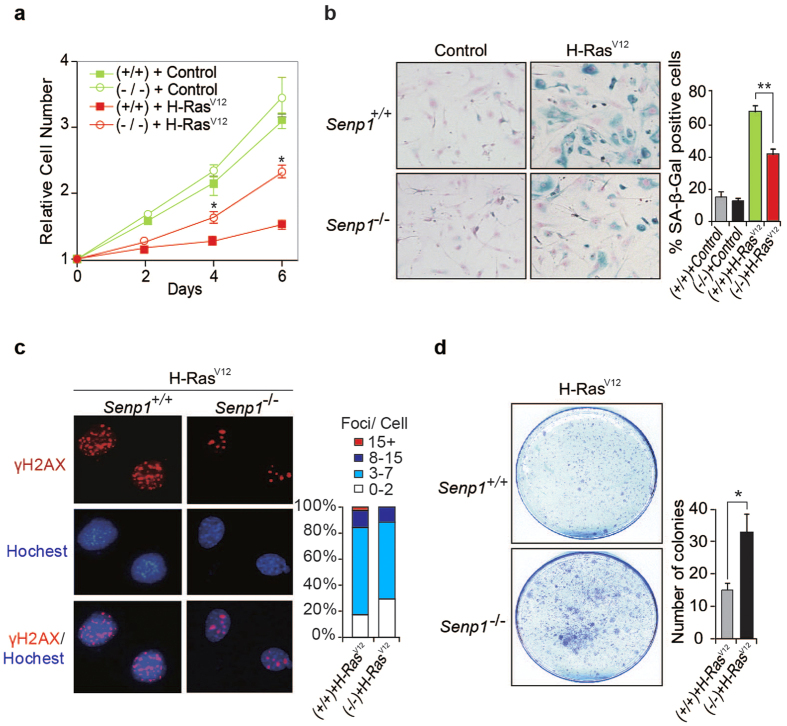
*Senp1*^−/−^ MEF cells are resistant to H-Ras^V12^-induced senescence. (**a**) Cell numbers were measured in primary *Senp1*^+/+^ and *Senp1*^−/−^ MEF cells (passage 2 or 3) infected with mock or H-Ras^V12^ lentiviral vectors. The cell numbers were counted for 6 days. Data represent the mean ± S.E.M. of three independent experiments. A p value of <0.05 (*) indicates a significant difference between H-Ras^V12^ lentiviral vectors-infected *Senp1*^+/+^ and *Senp1*^−/−^ MEFs. (**b**) Mock lentiviral vectors or H-Ras^V12^ lentiviral vectors-infected cells were stained for SA-β-gal (left panel); the percentage of SA-β-gal positive cells was counted at least in five fields (right panel) (**P < 0.01, Student’s t-test). (**c**) *Senp1*^+/+^ and *Senp1*^−/−^ MEF cells were stained for γH2AX (left panel); Percentage of cells with γH2AX foci per nucleus is shown in right panel. (**d**) Growth of *Senp1*^+/+^ and *Senp1*^−/−^ MEF cells at early-passage (passage 2 or 3) transduced with H-Ras^V12^ were measured by colony forming assay. Data represent the mean ±S.E.M. of three independent experiments (*P < 0.05, Student’s t-test).

Oncogene-induced senescence acts as a barrier to prevent oncogenic cell transformation and tumorigenesis^5^,^28^. To determine the role of SENP1 deficiency in oncogene-induced senescence, we stably transduced H-Ras^V12^ into *Senp1*^+/+^ or *Senp1*^−/−^ MEF cells at early-passages (passage 2 or 3). The cell proliferation assay showed that H-Ras^V12^ inhibited less cell growth in *Senp1*^−/−^ MEF cells than that in *Senp1*^+/+^ cells (Fig. 2a). The number of SA-β-gal positive cells was much less in H-Ras^V12^-transduced *Senp1*^−/−^ MEF cells than those in *Senp1*^+/+^ MEF cells (Fig. 2b). We also stained phosphorylated histone H2AX (γH2AX) and observed less γH2AX foci in H-Ras^V12^-transduced *Senp1*^−/−^ MEF cells when compared to *Senp1*^+/+^ MEF cells (Fig. 2c). Additionally, the percentages of γH2AX and macroH2A positive cells were also lower in H-Ras^V12^-transduced *Senp1*^−/−^ MEF cells than that in *Senp1*^+/+^ cells (Figure S1a–d). We further analysed the colony forming ability in H-Ras^V12^-transduced MEF cells. As shown in Fig. 2d, more colonies were observed in H-Ras^V12^-transduced *Senp1*^−/−^ MEF cells than in control cells. These data suggest that SENP1 deficiency can decrease oncogene-induced cell senescence.

### Downregulation of p19^Arf^ in *Senp1* ^−/−^ MEF cells

**Figure 3:**
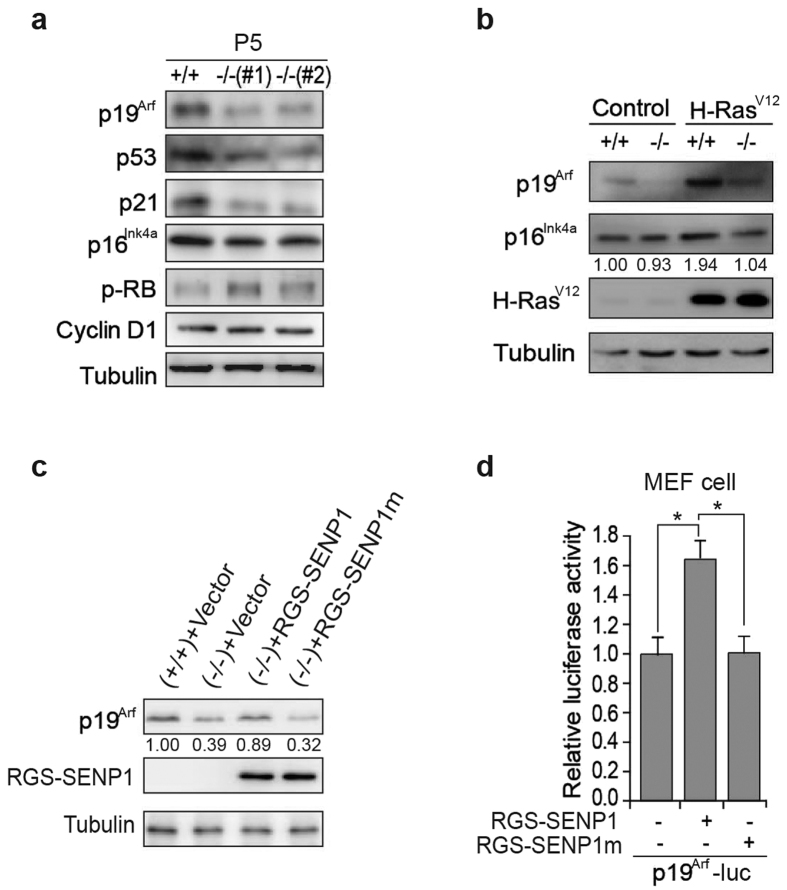
p19^Arf^/p53/p21 axis is down-regulated in *Senp1*^−/−^ MEF cells. (**a**) *Senp1*^+/+^ or *Senp1*^−/−^ MEF cells at passage 5 (P5) were analysed for expression of p19^Arf^, p53, p21, p16^Ink4a^, p-RB, and cyclin D1. The MEFs were cultured following a 3T3 protocol. Shown are the cropped blot images representing indicated proteins. Full-length blots are presented in the Supplementary Figure S3. (**b**) Expression of p19^Arf^ and p16^Ink4a^ was analysed in cell extracts from *Senp1*^+/+^ or *Senp1*^−/−^ MEF cells infected with H-Ras^V12^ lentiviral vectors. Cropped blot images shows the representing indicated proteins. Full-length blots are presented in the Supplementary Figure S4. (**c**) Primary *Senp1*^−/−^ MEF cells at passage 5 transfected with RGS-SENP1, RGS-SENP1m or Vector were analysed the expression of p19^Arf^ by western blotting. Shown are the cropped blot images representing indicated proteins. Full-length blots are presented in the Supplementary Figure S4. (**d**) P19^Arf^ transcription was analyzed in MEF cells transfected with p19^Arf^ luciferase plasmid and SENP1 or SENP1m plasmids. Data represent the mean ± S.E.M. of three independent experiments (*P < 0.05, Student’s t-test).

To understand the mechanism underlying SENP1 regulating senescence, we analysed the activity of the two major senescence-related signal pathways p19^Arf^/p53/p21 and p16^Ink4a^/Rb in *Senp1*^+/+^ and *Senp1*^−/−^ MEF cells. As shown in Fig. 3a, the expressions of p19^Arf^, p53, and p21 proteins were markedly decreased in *Senp1*^−/−^ MEF cells when compared to *Senp1*^+/+^ cells. However, p16^Ink4a^ and Rb proteins showed only mild reduction in *Senp1*^−/−^ MEF cells in comparison to *Senp1*^+/+^ cells. We also detected the expressions of p19^Arf^ and p16^Ink4a^ in H-Ras^V12^-transduced *Senp1*^+/+^ or *Senp1*^−/−^ MEF cells and found that H-Ras^V12^ significantly induced p19^Arf^ but not p16^Ink4a^ expression in both cells (Fig. 3b). The expression of p19^Arf^ was less increased in H-Ras^V12^-transduced *Senp1*^−/−^ MEFs when compared to *Senp1*^+/+^ cells. We further transfected SENP1 wild-type (SENP1 WT) or SENP1 catalytic mutant (SENP1m) in *Senp1*^−/−^ MEF cells and found that re-expression of SENP1 WT but not SENP1m could rescue p19^Arf^ expression in *Senp1*^−/−^ MEF cells (Fig. 3c). To further confirm the role of SENP1 in regulating p19^Arf^ expression, we constructed p19^Arf^ promoter-driven luciferase reporter gene and showed that SENP1 WT but not SENP1m could induce p19^Arf^ transcription (Fig. 3d). Taken together, these data reveal that SENP1 promotes p19^Arf^ expression.

### SENP1 promotes p19^Arf^ expression via de-SUMOylation of Bmi1

**Figure 4:**
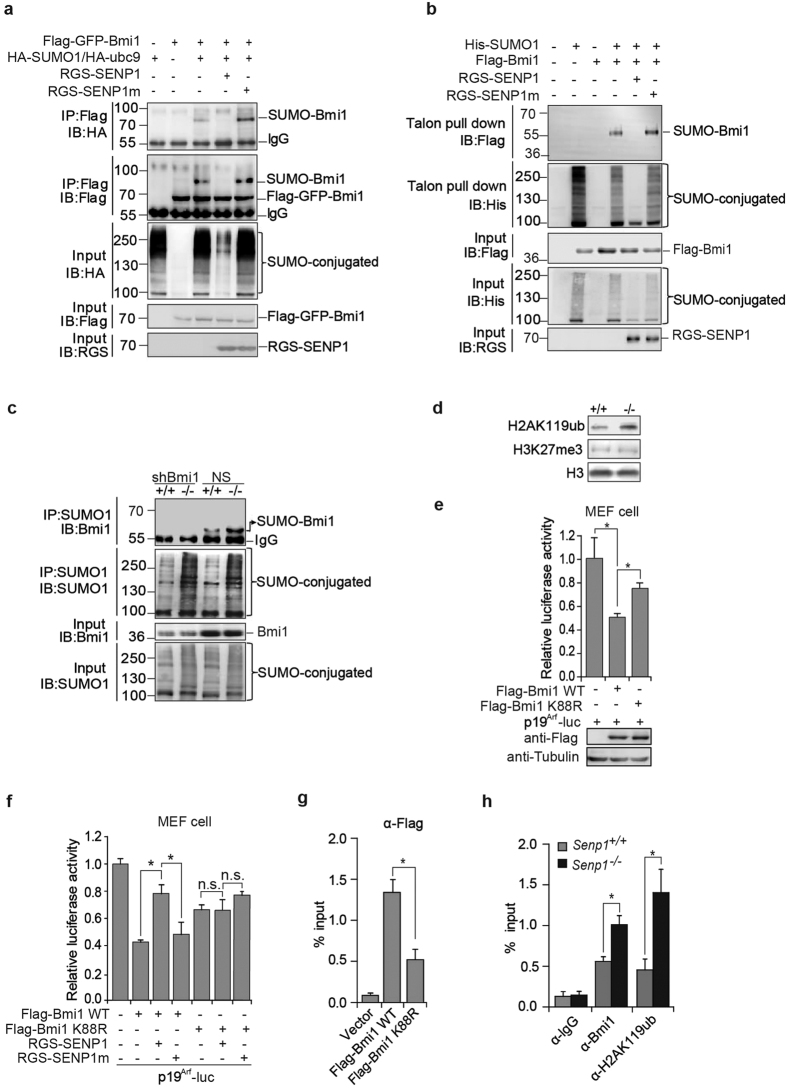
SENP1 de-SUMOylates Bmi1 and promotes p19^Arf^ expression. (**a**) Bmi1 was immunoprecipiated from cell lysates of 293T cells transfected with the indicated plasmids, and blotted for SUMO1 or Bmi1. Whole cell lysates were blotted with anti-HA, -Flag, or -RGS antibodies. Shown are the cropped blot images representing indicated proteins. Full-length blots are presented in the Supplementary Figure S5. (**b**) 293T cells were transfected with indicated plasmids. SUMO1-conjugated proteins were pulled down by talon beads from cell lysate. Bound proteins were blotted with anti-His, -Flag, or -RGS antibodies. Shown are the cropped blot images representing indicated proteins. Full-length blots are presented in the Supplementary Figure S5. (**c**) *Senp1*^+/+^ and *Senp1*^−/−^ MEFs or Bmi1 silencing (shBmi1) MEFs were lysed. SUMO-conjugated proteins were immunoprecipitated with anti-SUMO1 and bound proteins were blotted with anti-SUMO1 or -Bmi1 antibodies. Whole cell lysates were blotted with anti-SUMO1 or -Bmi1 antibodies. Shown are the cropped blot images representing indicated proteins. Full-length blots are presented in the Supplementary Figure S5. (**d**) H2AK119ub was blotted in *Senp1*^+/+^ and *Senp1*^−/−^ MEFs. Shown are the cropped blot images representing indicated proteins. Full-length blots are presented in the Supplementary Figure S5. (**e**) P19^Arf^ transcription was analyzed in MEFs transfected with p19^Arf^ luciferase plasmid, Flag-Bmi1 WT or K88R plasmids. Data represent the mean ± S.E.M. of three independent experiments (*P < 0.05, Student’s t-test). (**f**) P19^Arf^ transcription was analyzed in MEFs transfected with p19^Arf^ luciferase plasmid, Flag-Bmi1 WT or K88R, plus SENP1 or SENP1m plasmid. Data represent the mean ± S.E.M. of three independent experiments (*P < 0.05, Student’s t-test; n.s., non-significant). (**g**) *Senp1*^−/−^ MEFs were transfected with Flag-Bmi1 WT, Flag-Bmi1 K88R, or vector as indicated. Chromatins prepared from these cells were subjected to IP with anti-Flag beads followed by Real-time PCR using primers for p19^Arf^ promoter. Data represent the mean ± S.E.M. of three independent experiments (*P < 0.05, Student’s t-test). (**h**) Chromatins from *Senp1*^+/+^ and *Senp1*^−/−^ MEFs were subjected to IP with anti-Bmi1, -H2AK119ub or IgG followed by Real-time PCR using primers for p19^Arf^ promoter. Data represents the mean ± S.E.M. of three independent experiments (*P < 0.05, Student’s t-test).

Bmi1 is a critical negative regulator for p19^Arf^ and p16^Ink4a^ expression^23^,^29^,^30^. It has also been reported as a SUMOylated protein^27^. Therefore, we postulated that SENP1 might regulate p19^Arf^ expression via de-SUMOylation of Bmi1. To test whether SENP1 directly de-SUMOylates Bmi1, Flag-Bmi1 and HA-SUMO1 were co-expressed with RGS-tagged SENP1 or SENP1m in 293T cell lines. As a result, the co-expression of SENP1, not SENP1m, could de-conjugate SUMOylated Bmi1, while the SENP1m increase the SUMOylated Bmi1 (Fig. 4a,b) through a dominant negative activity to block the de-SUMOylation activity of endogenous SENP1^31^. More importantly, we observed that the SUMOylated Bmi1 proteins were accumulated in *Senp1*^−/−^ MEF cells (Fig. 4c). To exclude the possibility that Bmi1 expression was affected by SENP1, we checked the expression of Bmi1, as well as the expressions of other PRC1 components CBX4 and Ring1B, and found no changes in *Senp1*^−/−^ MEF cells (Figure S2A). These results indicate that SENP1 is a specific protease to de-SUMOylate Bmi1.

We further assessed whether SENP1 could affect Bmi1 ubiquitin ligase activity in mono-ubiquitination of histone 2A at K119. As shown in Fig. 4d, mono-ubiquitination of H2AK119 was increased in *Senp1*^−/−^ MEFs when compared to *Senp1*^+/+^ cells, suggesting that SENP1 mediated de-SUMOylation decreases Bmi1 ubiquitin ligase activity. We also measured the suppressive activity of Bmi1 WT and Bmi1 SUMOylation mutant (K88R) using p19^Arf^ promoter-driven luciferase assay. Bmi1 WT significantly reduced p19^Arf^ transcription, whereas Bmi1 K88R mutant showed a weaker repressive activity than Bmi1 WT (Fig. 4e). Meanwhile we found that SENP1, but not SENP1m could counteract the suppressive activity of Bmi1 WT on p19^Arf^ transcription (Fig. 4f).

We reasoned that SUMOylation might promote Bmi1 binding to p19^Arf^ promoter. To test it, we used chromatin immunoprecipitation (ChIP) assay to demonstrate that the occupancy of Bmi1 WT on p19^Arf^ promoter was much higher than that of Bmi1 K88R mutant (Fig. 4g). We also illustrated that Bmi1 occupancy on p19^Arf^ promoter was more profound in *Senp1*^−/−^ MEFs than that in WT cells, and that the ubiquitination of H2A at K119 of p19^Arf^ promoter was also increased in *Senp1*^−/−^ MEFs when compared to WT cells (Fig. 4h). Taken together, these data suggest that SENP1 promotes p19^Arf^ expression via de-SUMOylation of Bmi1.

### SUMOylation is essential for Bmi1 repression of senescence

**Figure 5:**
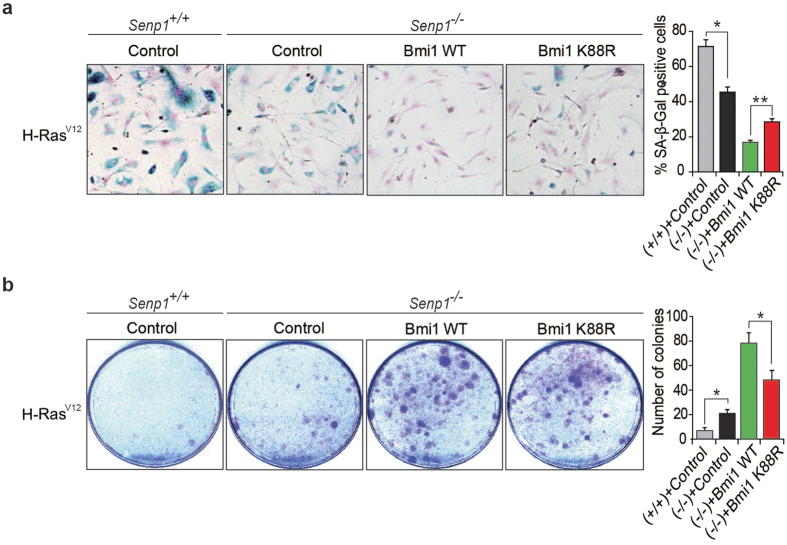
SUMOylation modulates Bmi1-mediated repression of cellular senescence. (**a**) H-Ras^V12^ transduced Bmi1 (WT or K88R)-*Senp1*^−/−^ MEF cells were stained for SA-β-Gal activity. Primary MEF cells at passage 2 or 3 were sequentially transduced with Bmi1 and H-Ras^V12^. The assay was performed at around passage 8. Representative images are shown in left panel. SA-β-Gal positive cells were counted at least in five fields (right panel). Data represent the mean ± S.E.M. of three independent experiments (*P < 0.05, Student’s t-test). (**b**) Growth of Bmi1 (WT or K88R)-*Senp1*^−/−^ MEF cells transduced with H-Ras^V12^ was measured by colony forming assay. Data represent the mean ± S.E.M. of three independent experiments (*P < 0.05, Student’s t-test).

To determine whether SUMOylation affect Bmi1 repression of cell senescence, we transduced H-Ras^V12^-*Senp1*^−/−^ MEF cells either with Bmi1 WT or K88R mutant. SA-β-gal staining showed that both Bmi1 WT and Bmi1 K88R expressions decreased the percentage of SA-β-gal positive cells in H-Ras^V12^-*Senp1*^−/−^ MEF cells compared to vector control (Fig. 5a). However, more SA-β-gal positive cells were observed in Bmi1 K88R-transduced cells than that in Bmi1 WT control. We further found that both Bmi1 WT and Bmi1 K88R expressions increased colony formation in H-Ras^V12^-*Senp1*^−/−^ MEF cells compared to vector control. Less colony were found in Bmi1 K88R-transduced H-Ras^V12^-*Senp1*^−/−^ MEF cells in comparison to Bmi-1 WT control (Fig. 5b). These data suggest that SUMOylation is essential for Bmi1 repression of cell senescence.

### SENP1 expression is associated with cell senescence in prostate PIN lesion

**Figure 6:**
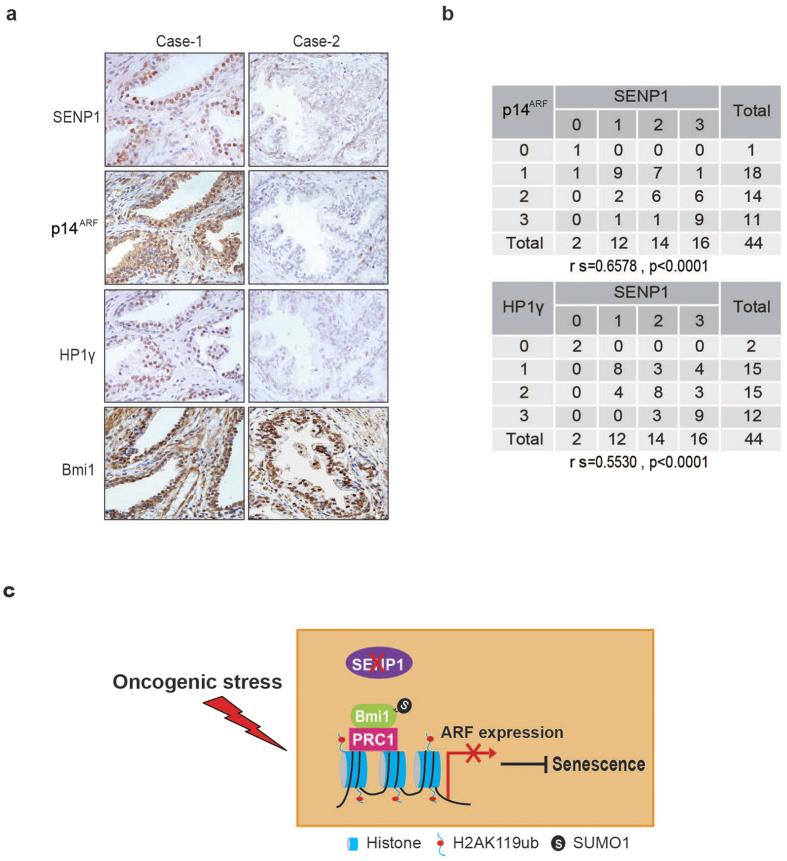
SENP1 is positively correlated with senescence marker in human PIN lesion. (**a**) Human PIN samples stained for SENP1, p14^ARF^, HP1γ and Bmi1. (**b**) Immunohistochemistry was performed to evaluate SENP1, p14^ARF^ and HP1γ expression on PIN TMA slides from patients. SENP1, p14^ARF^ and HP1γ protein level in these samples was scored from 0 to 3 based on the stained area percentage and immunostaining intensity. Spearman correlation coefficient was performed to evaluate the association of SENP1 with p14^ARF^ or HP1γ. (**c**) Working model shows the mechanism of SENP1 in regulating cell senescence.

Previously, we have shown that SENP1 was overexpressed in prostrate intraepithelial neoplasia (PIN) lesion^32^ and tumor cells^33^,^34^. Overexpression of SENP1 in mouse prostate induced PIN lesions, but not tumor formation^33^. We speculated that overexpression of SENP1 could promote the growth of prostate epithelial cell as well as cell senescence in PIN lesion. We thus collected 44 human prostate PIN samples and analysed the expression of SENP1, senescence markers heterochromatin proteins 1 γ (HP1γ)^35^,^36^, p16^INK4a^, p14^ARF^ and Bmi1 by immunohistochemistry staining in these samples. A score indicating SENP1, HP1γ, p16^INK4a^, p14^ARF^ or Bmi1 was given ranging from 0 to 3 based on the percentage of the stained area and immunostaining intensity in these samples. As shown, SENP1 expression was positively related to HP1γ (rs = 0.553, *P* < 0.0001), p14^ARF^ (rs = 0.6578, *P* < 0.0001), and p16^INK4a^ (rs = 0.6755, *P* < 0.0001) expressions in human PIN samples (Fig. 6a,b) (Figure S3a,b). However, it was not related to Bmi1 expression (rs = −1501, *P* = 3309) (Figure S3a,b). These data indicate that SENP1 expression is associated with cell senescence in prostate PIN lesion.

## Discussion

In this study, we demonstrate a role of SENP1 in cell senescence. SENP1 de-SUMOylates Bmi1 and reduces the occupancy of Bmi1 on p19^Arf^ promoter, leading to p19^Arf^ expression and cell senescence (Fig. 6c). SUMOylated Bmi1 was accumulated in *Senp1*^−/−^ MEFs (Fig. 4c) and SUMOylation promoted Bmi1 occupancy on p19^Arf^ promoter to suppress p19^Arf^ expression (Fig. 4f–h). Interestingly, *Bmi1*^−/−^ cells showed increased Ink4a/Arf expression, especially p16^Ink4a^ expression^23^, whereas p16^Ink4a^ expression is only mildly decreased in *Senp1*^−/−^ MEFs compared to p19^Arf^. These results suggest that SUMOylation might selectively modulate Bmi1 effect on p19^Arf^ by promoting its binding to the promoter locus, although currently it is unknown how SUMOylation modulates Bmi1 selection.

We showed the deficiency of SENP1 delays MEF cell senescence, which seems to contradict with the previously report on SENP1 represses cell senescence in human fibroblasts^37^. Yates *et al.* showed that shSENP1 in human fibroblasts induced senescence. They further showed that shSENP1 increased p53 expression via unknown mechanism. In the present study, we find that SENP1 deficiency reduces p19^Arf^/p16^Ink4a^/p53 expression via Bmi1. As mouse cells have very long telomeres (40–60 kb) when compared to human cells (5–15 kb)^38^, we currently don’t know whether this difference would contribute to their different responses to senescence. We also note another difference in the approaches that we have utilized for SENP1 deficiency from Yates *et al.*^37^. We deleted *Senp1* gene in genome of *Senp1*^−/−^ MEF cells, whereas SENP1 was silenced at mRNA level by shSENP1in HFF cells, which might cause off target effect.

The SUMOylated Bmi1 was first reported to accumulate at the DNA damage sites^27^. Our data showed the SUMOylation increased Bmi1 binding to DNA. It is possible the increased Bmi1 may recruit some DNA repairing proteins to repair oncogene induced DNA damage and to reduce the γH2Ax foci. Bmi1 is proposed to bind and increase Ring1B’s E3 ligase activity^39^. Bmi1/Ring1b, an autoinhibited RING E3 ubiquitin ligase, was also reported to promote SENP1 ubiquitination and degradation^40^, suggesting that this regulation might be a negative feedback mechanism to control SENP1 action on Bmi1, which would promote cell to be transformed during tumorigenesis. However, we don’t know whether SUMOylated Bmi1 further increases the E3 ligase activity of Ring1B and thereby affects ubiquitination of SENP1.

It is well-known that ROS-induced DNA damage can increase p53 activation, which in turn leads to cellular senescence^38^,^41^,^42^,^43^. Previously, we have shown that ROS production decreases in *Senp1*^−/−^ MEF cells because of the defect in mitochondrial biogenesis^44^. Thus, the lower ROS production might partially contribute to the reduction of senescence in *Senp1*^−/−^ MEF cells. It is evident that we observe a decrease in DNA damage marker γH2AX foci in *Senp1*^−/−^ MEF cells.

We previously found SENP1 was overexpressed in PIN lesion^32^ and tumor cells^33^,^34^. We also showed that overexpression of SENP1 in mouse prostate induced PIN lesions, but no tumor formation in the transgenic model^33^. We show herein that SENP1 expression is associated with the senescence in PIN lesion. We expect to see if bypassing senescence would promote PIN to cancer in the SENP1 prostate transgenic model. This is supported by our recent observations that tumor suppressor PTEN deficiency promote cancer development in SENP1 prostate transgenic mice partially through the decrease of p53 expression and cell senescence (unpublished data), suggesting the role of SENP1-mediated senescence in tumorigenesis, which need to be further explored in future.

## Material and Methods

### Mouse embryo fibroblasts isolation and cell culture

All animal procedures were approved by the Institutional Animal Care and Use Committee of Shanghai Jiao Tong University School of Medicine, which were carried out in accordance with the guidelines (IACUC No. 2013027). The generation of *Senp1*^+/+^ or *Senp1*^−/−^ MEF cells was previously described^45^. MEF cells were cultured in completed DMEM containing 10% FBS at 37 °C under 5% CO_2_. For replicative senescence assay, cells were passaged following a 3T3 protocol^46^,^47^. Cells were split every 3 days and replanted at a density of 1 × 10^6^ cells per 10 cm dish for 20 passages. For colony forming assay, MEF cells were seeded at a density of 5 × 10^4^ cells per 10 cm dish and cultured for 2 weeks. Then cells were washed with phosphate-buffered saline, fixed in 70% ethanol for 15 min and stained with crystal violet. Colonies were observed under microscope. The cell mass with more than 50 cells was counted as a colony.

### Plasmids and antibodies

Flag-Bmi1 (WT or K88R) and Flag-GFP-Bmi1 (WT or K88R) were generated using standard cloning procedures. K88R mutation was introduced into Bmi1 by site directed mutagenesis using QuikChange Site-Directed Mutagenesis Kit (Agilent California, USA). HA-SUMO1, HA-UBC9, RGS-SENP1 and RGS-SENP1m were previously described^45^,^48^. Anti-FLAG (mouse; #F1804) and anti-HA(mouse; #H3663) antibodies were from Sigma; anti-RGS-his (mouse; #34610) antibody from QIAGEN; anti-H-Ras (mouse; #sc-53959) and p16^Ink4a^ (mouse; #sc-1661) antibodies from Santa Cruz; anti-SUMO1 (rabbit; #ab5316), H3K27me3 (mouse; #ab6002) and p19^Arf^ (rabbit; #ab80) antibodies from Abcam; anti-Bmi1 (mouse; #05–637), γH2AX (mouse; #05–636) and uH2AK119 (mouse; #05–678) antibodies from Millipore.

### Senescence associated β-galactosidase staining

MEF cells were plated at a density of 1 × 10^5^ cells in 35 mm dishes. Cells were fixed and stained according to the instructions supplied by the senescence β-galactosidase staining kit (Beyotime biotechnology, China). Briefly, cells were washed with PBS and fixed in SA-β-Gal fixing solution for 15 min at room temperature. Cells then were washed 3 times with PBS and stained with working solution (10 μl buffer A, 10 μl buffer B, 930 μl buffer C, and 50 μl X-Gal solution) overnight at 37 °C. Cells were counterstained with nuclear fast red. The population of SA-β-Gal positive cells was determined by counting 400 cells in at least 5 fields per dish and images were taken using a phase-contrast microscope at 400× magnification (Olympus, Japan). The proportions of cells positive for SA-β-Gal activity are shown as the percentage of the total number of cells counted in each dish.

### BrdU incorporation assay

Sub-confluent *Senp1*^+/+^ and *Senp1*^−/−^ MEF cells at passage 5 (P5) and passage 12 (P12) were labelled for 4 h with 10 μM 5-bromo-2′-deoxyuridine (BrdU; Amersham). Labelled MEF cells were planted on cover slips and fixed in 5% acetic acid and 95% ethanol for 15 min at −20 °C. After fixation, cells were washed with PBS and treated with 0.1 mg/ml RNase A for 30 min at 37 °C. After washed with PBS, cells were washed with 2 N HCl, 0.5% Triton X-100 for 30 min at room temperature. Thereafter the cells were washed with PBS and incubated with BrdU antibody and FITC conjugated secondary antibody for 1 h. After washing with PBS, cells were counterstained with DAPI and analyzed under fluorescence microscope.

### Immunoprecipitation and immunoblotting

Cells were washed once and lysed in the presence of 10 mM N-ethylmaleimide (NEM) using ice-cold RIPA buffer (50 mM Tris-HCl (pH 7.4), 400 mM NaCl, 1% Triton X-100, 0.1% SDS, 1 mM PMSF, and protease inhibitors) for 30 min on ice. Cell lysates were centrifugated at 14,000 × g for 10 min at 4 °C. The supernatants were collected and incubated with appropriate antibodies overnight at 4 °C, followed by incubation with protein A/G-Sepharose beads (Amersham Biosciences) for another 2 h at 4 °C. After washing three times with RIPA buffer, the immunoprecipitates were eluted with Laemmli sample buffer, elute boiled, and subjected to western blot analysis.

### Immunofluorescent staining

Paraformaldehyde-fixed cells were treated with 0.3% Triton X-100 in PBS for 15 min. After washing with PBS, cells were blocked with 1% BSA in PBS for 1 h, and then incubated with anti-γH2AX(1:200) antibody overnight at 4 °C, followed by incubation with APC conjugated secondary antibody(1:200) for 1 h. Cells were then counterstained with DAPI. Samples were visualized with an Olympus fluorescence microscopy.

### Chromatin immunoprecipitation (ChIP) assays

Formaldehyde-crosslinked chromatin was prepared from MEF cells and immunoprecipitations were performed using the ChIP Assay Kit according to the manufacturer’s recommended protocol (Upstate Biotechnology). Antibodies used for ChIP assays were anti-Flag-M2, anti-Bmi1, anti-H2AK119ub or IgG. A pair of real-time PCR primers was used for amplification of the promoter segments of the mouse p19^Arf^ gene as previously^49^: forward, 5′-AAAACCCTCTCTTGGAGTGGG-3′; reverse, 5′-GCAGGTTCTTGGTCACTGTGAG-3′.

### Luciferase assays

P19^Arf^ promoter containing 1202 nucleotides upstream the ATG start codon was cloned into the pGL3-basic luciferase reporter vector (Promega) using standard cloning procedures. For reporter assays, MEF cells were transfected with X-tremeGENE HP DNA Transfection Reagent (Roche Applied Science). 36 h after transfection, the luciferase was assayed using the Dual-Luciferase reporter assay system (Promega). Luciferase activity of the reporter construct was normalized on the basis of the *Renilla* luciferase activity.

### Statistical analyses

All data are represented by the mean ± S.E.M. of at least three independent experiments. GraphPad Prim 5 (GraphPad Software, La Jolla, CA) was used for statistical analysis. Data was analysed using two-tailed student’s t-test. A p value of <0.05 was considered significant.

## Additional Information

**How to cite this article**: Xia, N. *et al.* SENP1 Is a Crucial Regulator for Cell Senescence through DeSUMOylation of Bmi1. *Sci. Rep.* **6**, 34099; doi: 10.1038/srep34099 (2016).

## Supplementary Material

Supplementary Information
