# Supplementary material for: SENP1 Is a Crucial Regulator for Cell Senescence through DeSUMOylation of Bmi1
Source: Sci Rep. 2016 Sep 23;6:34099. doi: 10.1038/srep34099 (PMC5034316; doi:10.1038/srep34099)
Supplement: Supplementary Information [file srep34099-s1.pdf]

SENP1 Is a Crucial Regulator for Cell Senescence through De-SUMOylation of Bmi1

Nansong Xia[1,5], Juan Cai[1,5], Feifei Wang[1,5], Baijun Dong[2], Song Liu[3], Fengling Chen[4], Jinke Cheng[1]*, Yong Zuo[1]*

[1] Department of Biochemistry and Molecular Cell Biology, Shanghai Key Laboratory for Tumor Microenvironment and Inflammation, Shanghai Jiao Tong University School of Medicine, Shanghai, China .

[2] Department of Urology, Renji Hospital, Shanghai Jiao Tong University School of Medicine, Shanghai, China.

[3] Department of Respiratory Medicine, Xinhua Hospital, Shanghai Jiao Tong University School of Medicine, Shanghai , China.

[4] Shanghai Third People's Hospital, Shanghai Jiao Tong University School of Medicine, Shanghai , China.

[5] N Xia, J Cai, and F Wang contribute equally

* To whom correspondence should be addressed.

Tel: (86) 21 -64661525; Fax: (86) 21 -64661525; E -mail: jkcheng@shsmu.edu.cn

Present Address: Jinke Cheng, Department of Biochemistry and Molecular Cell Biology, Shanghai Jiao Tong University School of Medicine, 280 Chongqing South Road, Shanghai, 200025,China

Tel: (86) 21 -64661525; Fax: (86) 21 -64661525; E -mail: zuoyong @shsmu.edu.cn

Present Address: Yong Zuo , Department of Biochemistry and Molecular Cell Biology, Shanghai Jiao Tong University School of Medicine, 280 Chongqing South Road, Shanghai, 200025,China

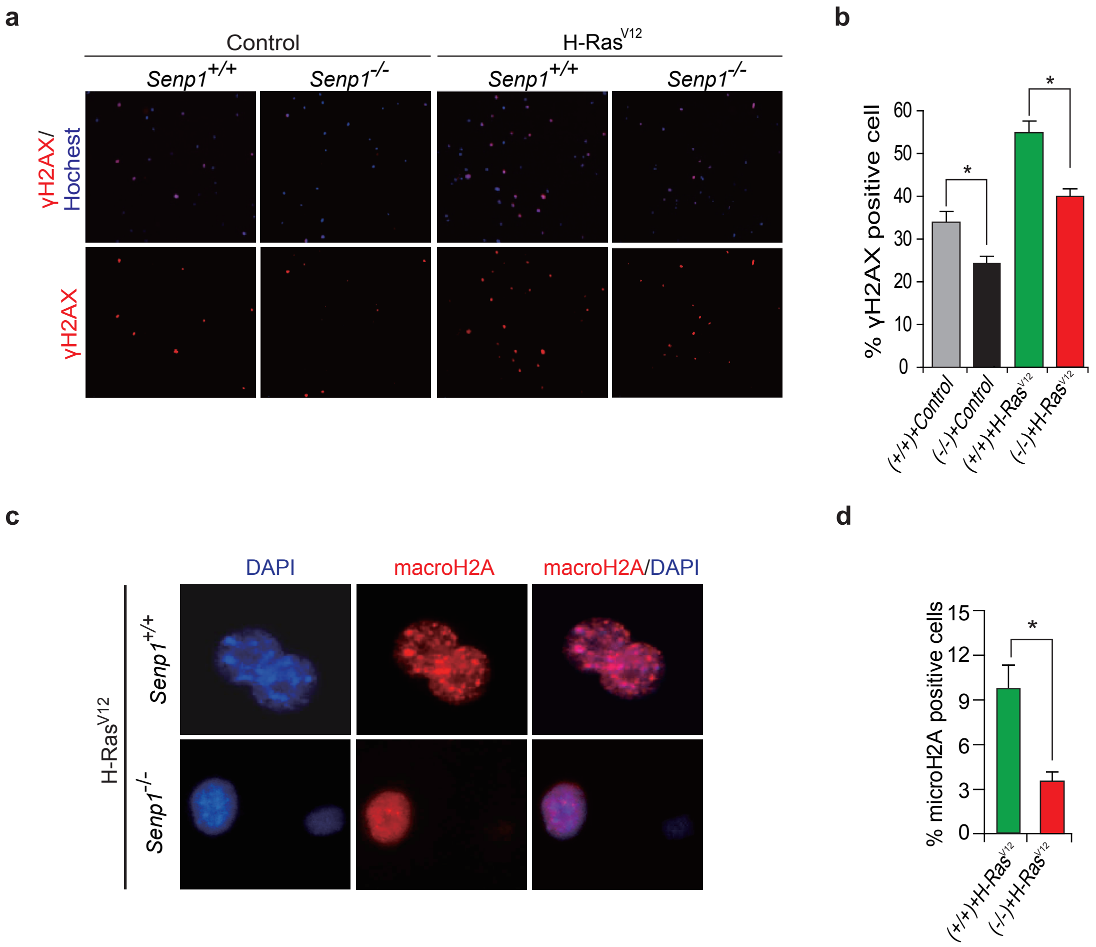

Figure S1: Less γH2AX staining in *Senp1*$^{-/-}$ MEF cells. (**a**) Representative images of γH2AX staining in H-Ras^{V12} transduced *Senp1*$^{+/+}$ and *Senp1*$^{-/-}$ MEF cells. (**b**) The percentage of γH2AX positive cells in H-Ras^{V12} transduced *Senp1*$^{+/+}$ and *Senp1*$^{-/-}$ MEF cells (* $p<0.05$, student's t-test). (**c**) Representative images of macroH2A staining in H-Ras^{V12} transduced *Senp1*$^{+/+}$ and *Senp1*$^{-/-}$ MEF cells. (**d**) The percentage of macroH2A positive cells in H-Ras^{V12} transduced *Senp1*$^{+/+}$ and *Senp1*$^{-/-}$ MEF cells (* $p<0.05$, student's t-test).

a

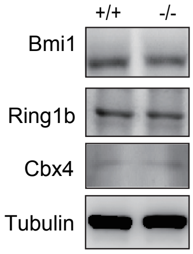

Figure S2: (**a**) The expressions of Bmi1, Ring1b and Cbx4 were determined by western blot in primary *Senp1* $^{+/+}$ and *Senp1*$^{-/-}$ MEF cells.Tubulin serves as a loading control.

a.

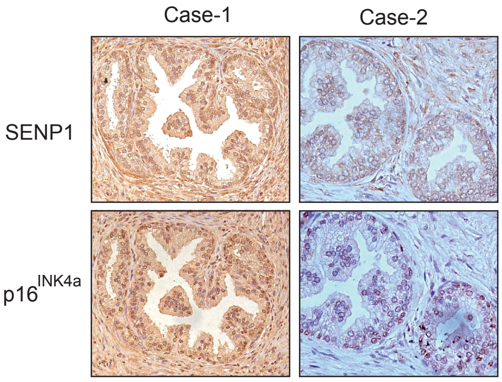

b.

p16^{INK4a}	SENP1				Total
	0	1	2	3	
0	0	2	0	0	2
1	2	7	4	2	15
2	0	2	9	2	13
3	0	1	1	12	14
Total	2	12	14	16	44

r s=0.6755 , p<0.0001

c.

Bmi1	SENP1				Total
	0	1	2	3	
0	0	0	0	2	2
1	0	0	3	3	6
2	2	5	3	4	14
3	0	7	8	7	22
Total	2	12	14	16	44

r s=-0.1501 ,p=0.3309

Figure S3: SENP1 is positively correlated with senescence marker p16^{INK4a} in human PIN lesion. (**a**) Representative human PIN samples stained for SENP1 and the senescence marker p16^{INK4a}. (**b**) and (**c**) SENP1, p16^{INK4a} and Bmi1 protein level in these samples were scored from 0 to 3 based on the stained area percentage and immunostaining intensity. Spearman correlation coefficient was performed to evaluate the association of SENP1 with p16^{INK4a} or Bmi1.

Figure 3a

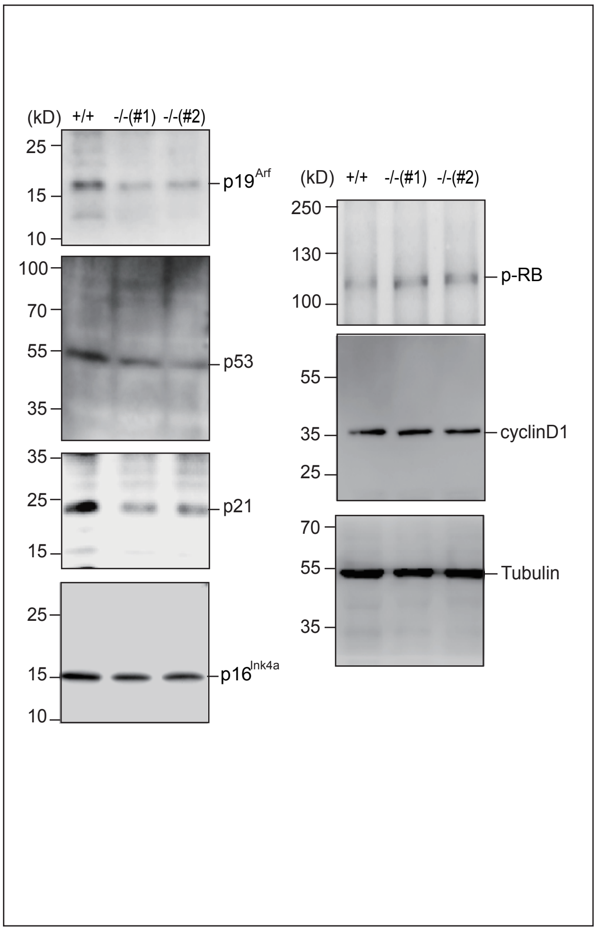

Figure 3b

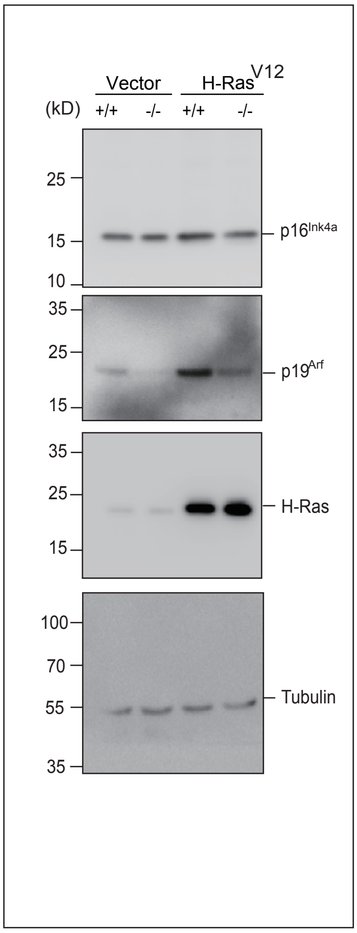

Figure 3c

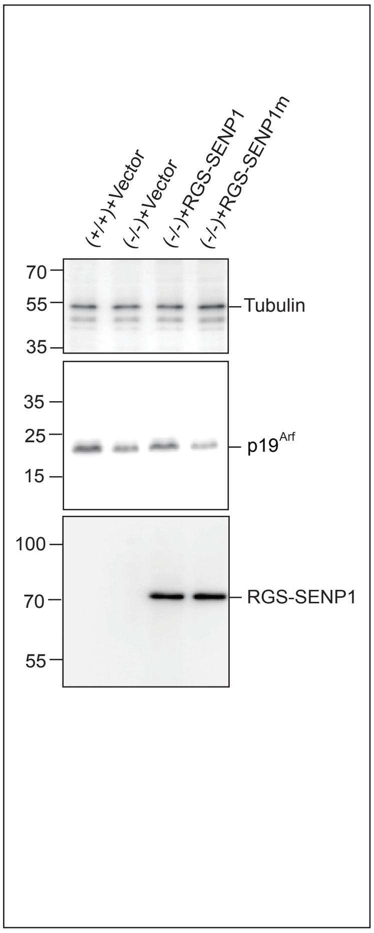

Figure S4: The full-length western blots of the figure 3.

Figure 4a

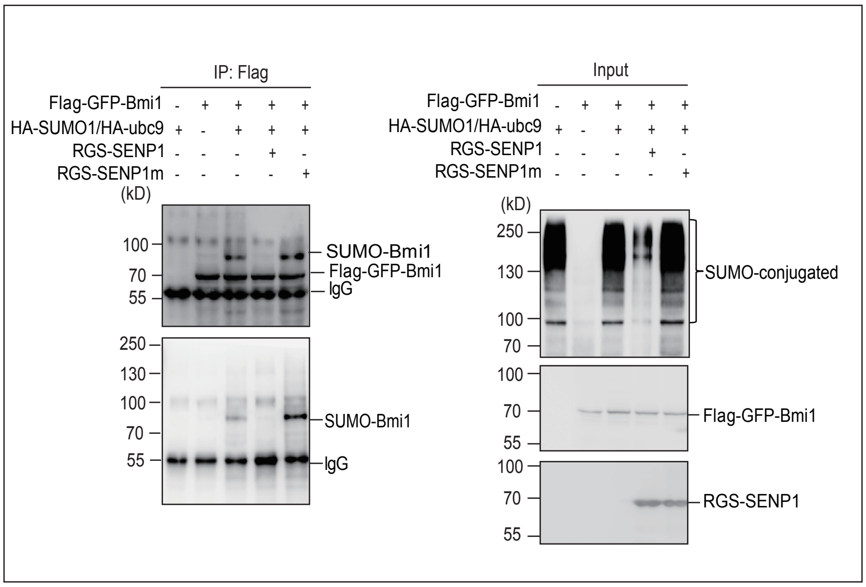

Figure 4b

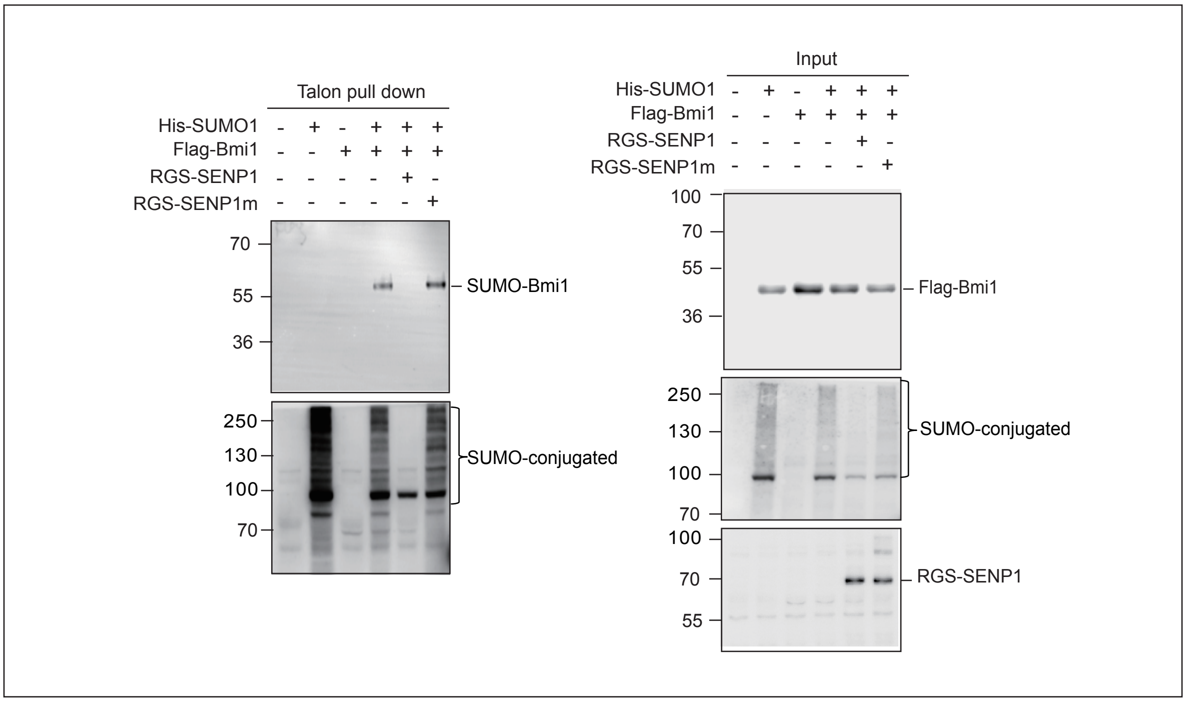

Figure 4c

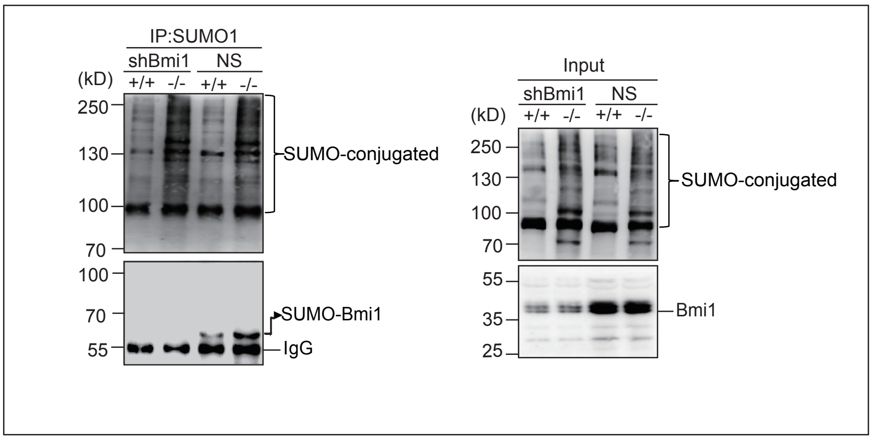

Figure 4d

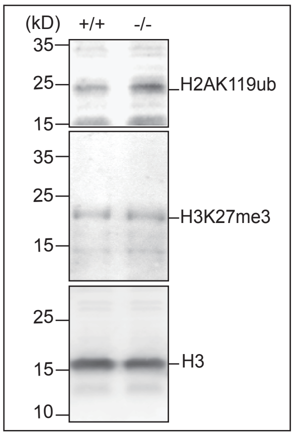

Figure S5: The full-length western blots of the figure 4.
